# Using the distance between sets of hierarchical taxonomic clinical concepts to measure patient similarity

**DOI:** 10.1186/s12911-019-0807-y

**Published:** 2019-04-25

**Authors:** Zheng Jia, Xudong Lu, Huilong Duan, Haomin Li

**Affiliations:** 1 College of Biomedical Engineering and Instrument Science, Zhejiang University, Hangzhou, China; 2 The Children’s Hospital, Zhejiang University School of Medicine, Hangzhou, China; 3 The Institute of Translational Medicine, Zhejiang University, Hangzhou, China

**Keywords:** Taxonomic concept, Patient similarity, Concept similarity, Predictive model, ICD-10, Data visualization

## Abstract

**Background:**

Many clinical concepts are standardized under a categorical and hierarchical taxonomy such as ICD-10, ATC, etc. These taxonomic clinical concepts provide insight into semantic meaning and similarity among clinical concepts and have been applied to patient similarity measures. However, the effects of diverse set sizes of taxonomic clinical concepts contributing to similarity at the patient level have not been well studied.

**Methods:**

In this paper the most widely used taxonomic clinical concepts system, ICD-10, was studied as a representative taxonomy. The distance between ICD-10-coded diagnosis sets is an integrated estimation of the information content of each concept, the similarity between each pairwise concepts and the similarity between the sets of concepts. We proposed a novel method at the set-level similarity to calculate the distance between sets of hierarchical taxonomic clinical concepts to measure patient similarity. A real-world clinical dataset with ICD-10 coded diagnoses and hospital length of stay (HLOS) information was used to evaluate the performance of various algorithms and their combinations in predicting whether a patient need long-term hospitalization or not. Four subpopulation prototypes that were defined based on age and HLOS with different diagnoses set sizes were used as the target for similarity analysis. The F-score was used to evaluate the performance of different algorithms by controlling other factors. We also evaluated the effect of prototype set size on prediction precision.

**Results:**

The results identified the strengths and weaknesses of different algorithms to compute information content, code-level similarity and set-level similarity under different contexts, such as set size and concept set background. The minimum weighted bipartite matching approach, which has not been fully recognized previously showed unique advantages in measuring the concepts-based patient similarity.

**Conclusions:**

This study provides a systematic benchmark evaluation of previous algorithms and novel algorithms used in taxonomic concepts-based patient similarity, and it provides the basis for selecting appropriate methods under different clinical scenarios.

**Electronic supplementary material:**

The online version of this article (10.1186/s12911-019-0807-y) contains supplementary material, which is available to authorized users.

## Background

An enormous volume of digitized clinical data is generated and accumulated rapidly since the widespread adoption of electronic medical records (EMRs). These massive quantities of data hold the promise for propelling healthcare’s evolution from a proficiency-based art to a data-driven science, from a reactive mode to a proactive mode, and from one-size-fits-all medicine to personalized medicine. Personalized medicine refers to tailoring medical treatment to the individual characteristics of each patient, which literally means the ability to classify individuals into subpopulations that differ in their susceptibility to a disease or their response to a specific treatment. In particular, the personalized medicine is based on the ability of quantitatively measuring the individual distances between patients in a population.

Patient similarity analytics focus on homogeneous cohorts within heterogeneous patient populations [1]. With a patient similarity measure in place, many advanced applications can be enabled, such as improving the precision of predicting a curative effect [2], recommending therapy [3], identifying the relationship between diseases and co-occurrence [4], and aiding clinical decision-making at the point-of-care. In this way, patient similarity represents a paradigm shift that introduces innovation to optimize the personalization of patient care.

A challenge of patient similarity analysis is to identify the appropriate and effective usage of the non-numerical clinical concepts in EMR to derive a similarity measure between pairwise patients. Consider a case where patient A has postrheumatic arthropathy (M12.0), patient B has rheumatoid arthritis (M06.9) and patient C has rheumatic arthritis (I00.x01). The simple word-based similarity method regarding the consistency of the names of concepts cannot judge whether A is more similar to B or C. However, from a doctor’s point of view, A and B are obviously similar since both postrheumatic arthropathy and rheumatoid arthritis are inflammatory polyarthropathy whereas rheumatic arthritis is a type of acute rheumatic fever. Fortunately, many clinical concepts have been classified in hierarchical taxonomies such as ICD-10, ICD-9-CM-3, and ATC, which encode diseases, procedures, and drugs respectively [5]. The UMLS also provides a semantic network to integrate millions of concepts represented in the UMLS Metathesaurus. The hierarchical structure implies semantic relationships and distances between concepts. Therefore, measuring taxonomic concept similarity is a generic issue in patient similarity analysis.

In the recent past, many clinical concepts such as diagnoses [3, 6–9], symptoms [8, 10], demographics [7, 8, 10], health behavior [10], laboratory tests [3, 7, 10], signs [8], procedures [3] and drugs [3, 6, 7, 10] have been used to measure patient similarity and support clinical decisions. Among these studies, measuring taxonomic concept similarity has been indispensable. However, there is still no consistent conclusion regarding the best approach to assess the similarity between patients or subpopulations, which are usually modeled as a set of concepts. Therefore, the choice of the most appropriate algorithm at different levels and under various clinical scenarios is still a challenge.

### Related work

**Fig. 1:**
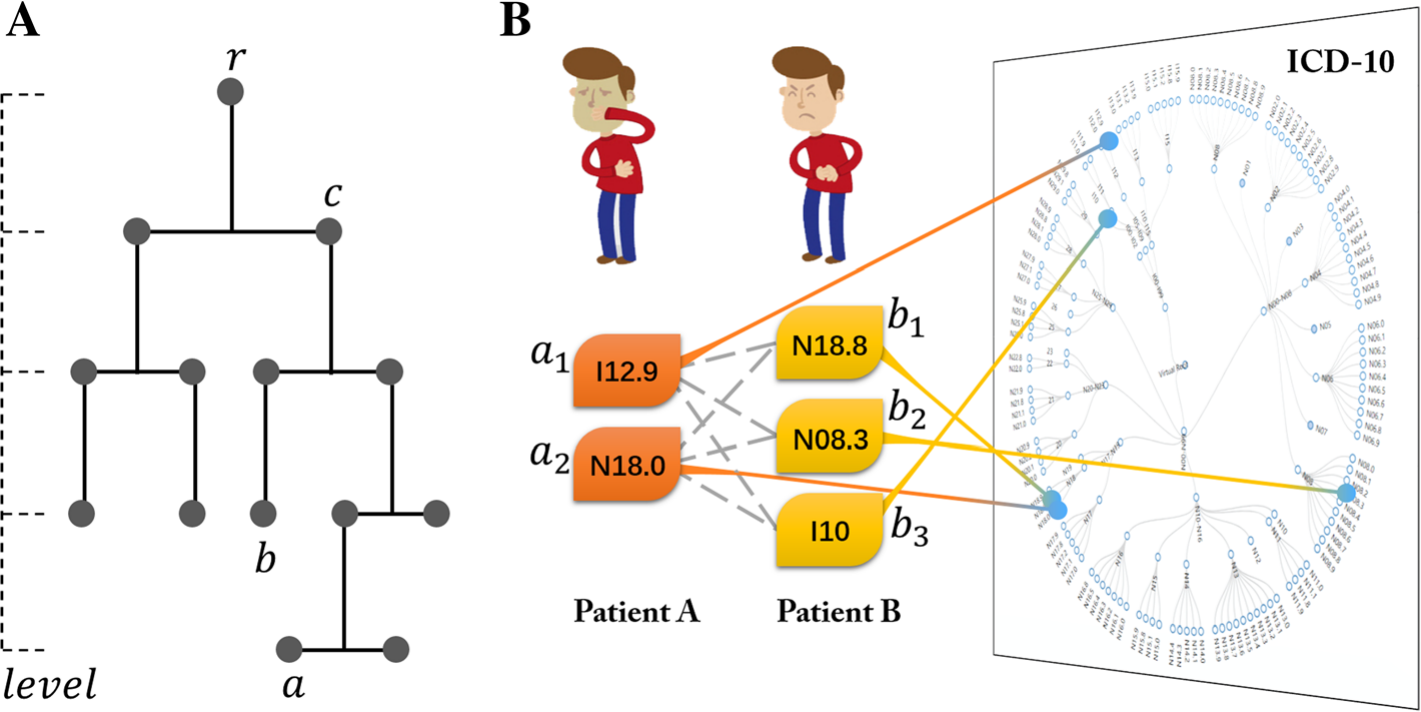
Taxonomic clinical concepts and patient similarity. **a** Taxonomic concepts and concepts semantic similarity. **b** Patient similarity based on the concept set-level similarity

Taxonomic concepts imply semantic relationships and distances. As shown in Fig. 1a, taxonomic concepts are usually organized hierarchically. Intuitively, concepts under the same branch will be more similar than concepts from different branches. Generally, the semantic similarity [11, 12] between two taxonomic concepts can be measured by two approaches [13]: the probabilistic approach and the information-theoretic approach. Probabilistic approaches are traditional data-driven methods proposed for categorical data and they address the frequency distribution of the concept in the patient set. Information-theoretic approaches consider the information content (IC) of concepts. The IC of a concept is a fundamental dimension stating the amount of embedded information in computational linguistics [14, 15]. Concrete and specialized entities in a discourse are generally considered to present more IC than general and abstract ones. Boriah [13] proved that the information-theoretic approach performs better than the probabilistic approach when explaining observed groups in clinical data. In this paper, we restrict out discussion to the information-theoretic approaches.

There are many approaches to calculate the IC of a taxonomic concept. A simple way is to assign different IC values to different levels of concepts (as shown in Table 1 IC #1 Formula). Therefore, a specific concept has a higher IC value than a general concept. Considering ICD-10 as an example, the IC of a virtual root is 1, then, the IC of a chapter of ICD is 2, and so on, so that the IC of the full range of ICD expansion nodes is 5 [16]. The other more complicated ontology-based IC computation model is proposed by Sanchez [14]. As shown in Table 1 IC #2 Formula, this method calculates the IC of a concept depending on the count of taxonomic leaves of a concept’s hyponym tree (|*leaves*(*a*)|) and the number of taxonomic subsumers (|*subsumers*(*a*)|).

**Table 1:** The formula used in the taxonomic concept-based patient similarity

	#	Formula		Reference
Information Content (IC)	1	levels(a → r)		[13]
	2	$-\log \left(\frac{\frac{\left| leaves(a)\right|}{\left| subsumers(a)\right|}+1}{\left| leaves(r)\right|+1}\right)$		[14]
Code-level Similarity (CS)	1	$$ \left\{\begin{array}{c}0,\kern0.5em if\ a=b\\ {}1,\kern0.5em otherwise\end{array}\right. $$		–
	2	$1-\frac{2 IC(c)}{IC(a)+ IC(b)}$		[16, 23]
	3	$1-{e}^{\alpha \left( IC(a)+ IC(b)-2 IC(c)\right)}\bullet \frac{e^{\beta IC(c)}-{e}^{-\beta IC(c)}}{e^{\beta IC(c)}+{e}^{-\beta IC(c)}}$		[17]
	4	$\frac{IC(l)- IC(c)}{IC(l)}$		–
Set-level similarity (SS)	1	Dice	$1-\frac{2\mid A\cap B\mid }{\mid A\mid +\mid B\mid }$	–
	2	Jaccard	$1-\frac{\mid A\cap B\mid }{\mid A\cup B\mid }$	–
	3	Cosine	$1-\frac{\mid A\cap B\mid }{\sqrt{\mid A\mid \bullet \mid B\mid }}$	–
	4	Overlap	$1-\frac{\mid A\cap B\mid }{\min \left\{|A|,|B|\right\}}$	–
	5	$\frac{1}{\mid A\mid +\mid B\mid}\bullet \left(\sum \limits_{a\in A}\underset{b\in B}{\min } CS\left(a,b\right)+\sum \limits_{b\in B}\underset{a\in A}{\min } CS\left(b,a\right)\right)$		[17]
	6	$\frac{1}{\mid A\cup B\mid}\bullet \left(\sum \limits_{a\in A\setminus B}\frac{1}{\mid B\mid}\sum \limits_{b\in B} CS\left(a,b\right)+\sum \limits_{b\in B\setminus A}\frac{1}{\mid A\mid}\sum \limits_{a\in A} CS\left(b,a\right)\right)$		[18]

With the IC of concepts, there are several ways to measure the similarity of two concepts. Four representative code-level similarity (CS) formulas are listed in Table 1. For the sake of notation, *a* and *b* are two concepts such that their similarity will be measured, as shown in Fig. 1a. *c* is defined as the least common ancestor (LCA) of *a* and *b* in the taxonomy. *r* and *l* represent the root and the total levels in the taxonomy, respectively. The CS #1 Formula, which is a binary similarity judgment, is efficient and simple to implement but cannot provide enough discrimination power in many applications. The CS #2 Formula is based on the information-theoretic definition of similarity proposed by Wu [16]. The CS #3 Formula by Li [17] introduced two parameters to scale the contribution of the IC of LCA and the IC of two concepts. On a benchmark data set, the author obtained the optimal parameters settings as α = 0.2 and β = 0.6, respectively. The CS #4 Formula is a simplified form of CS #2 Formula when *a* and *b* are in the deepest level. While it is not suitable when *a* and *b* are in other cases.

A patient usually suffers from multiple health problems and is diagnosed with a group of ICD codes, i.e., an ICD-10 set (as shown in Fig. 1b). The patient similarity is measured by the resemblance of two concept sets. Considering that A and B are two sets of taxonomic concepts, *a* is one of the concepts in A and *b* belongs to B. Six formulas to calculate set-level similarity (SS) are listed in.

Table 1. For the binary code-level similarity, some classical methods, such as Dice, Jaccard, Cosine, and Overlap, can be used to calculate set-level similarity. The other two formulas measure the resemblance of two concept sets through different approaches. The SS #5 Formula uses the most similar concept pair’s average value to represent the set-level similarity. The SS #6 Formula considers all the concept pair’s average similarity value as the set-level similarity.

ICD is a widely used taxonomy in clinical classification systems. Several patient similarity measures have been developed to detect similarity in patient records by referring to the ICD codes of diagnoses in the past few years. Gottlieb [7] used discharge ICD codes of past and current hospitalizations to construct a patient medical history profile to compute the similarity of patients. In Zhang’s research [6], the patient similarity was evaluated by the Tanimoto coefficient of co-occurring ICD-9 diagnosis codes. A novel distance measurement method for categorical values such as ICD-10 that takes the path distance between concepts in a hierarchy into account was proposed in Girardi’s research [18]. In Rivault’s research [19], diagnoses (ICD-10), drugs (ATC), and medical acts (CCAM) are used to reconstruct the care trajectories. The longest similar subsequence that accounts for the semantic similarity between events is proposed to compare medical episodes. However, all of these algorithms still lack a system evaluation. The strengths and weaknesses of various combinations of these algorithms under different clinical applications are not clear.

In different clinical scenarios, the taxonomic concept set sizes are different. According to the observation of clinical data in a one-year EMR dataset, the average number of distinct drugs used for each patient visit is approximately 13, and the average number of distinct diagnoses is approximately 8. However, the procedures during a patient visit may vary from tens to hundreds. Even when dressing the same taxonomy, there are different approaches to perform the patient similarity analysis. The patient-patient diagnosis similarity analysis addresses a relatively small set size. However, for patient-subpopulation or subpopulation-subpopulation diagnosis similarity analysis, the method may need to cope with different scenarios in which the concepts’ set sizes may be relatively large and unbalanced. Choosing appropriate formulas to measure the distance of concept sets to assist patient similarity analysis under different scenarios remains a challenge.

In this study, we create a more complicated clinical scenario for patient similarity analysis. The study cohort data were collected from the same nephrology department with basically similar conditions but with different complications. We systematically evaluated the previous algorithms and two new set-level similarity algorithms with different evaluation approaches, such as data visualization and the F-score measure of a specific prediction task.

## Methods

### Data set for this study

The EHR dataset that used for this study were obtained from a third-level grade-A hospital, Shanxi Dayi Hospital. The data sharing and utilization agreements were signed and approved by the IRB of Dayi Hospital (Project N.O. 2012AA02A601). The detail of data was interpreted in Additional file 1. The patient consent was waived, as utilization of anonymized history data does not currently require patient consent.

### Visual evaluation of the IC-level algorithms

A total of 433 distinct 4-character ICD-10 subcategories as interpreted in Additional file 1 are used to evaluate the algorithms to calculate IC of taxonomic concepts. The data set covers most common clinical problems in nephrology department. Two IC algorithms and the code-level similarity # 2 Formula were used to calculate the distance between each pair of these ICD-10 codes. Based on these results two distance matrices were generated. To visualize the structural information of distance matrices of ICD codes, a graph drawing tool is applied to implement the geometric representation of networks, named multi-dimensional scaling layout (MDS) [20]. It reduces the dimension from high space to low space as well as maintains the relative positions of individuals and well-organized layout. Different ICD-10 concepts were colored by their subcategories. The better IC algorithm can be expected to demonstrate a clear discrimination power between different ICD-10 subcategories.

### Set-level similarity algorithms

Two novel algorithms are proposed as shown below. The SS #7 Formula calculates all the concept pair’s average similarity values to present the set-level similarity using a different mean approach compared to SS #6 Formula.

$$ \frac{1}{\left|A\right|\bullet \left|B\right|}\bullet \sum \limits_{a\in A,b\in B} CS\left(a,b\right)\kern7.75em \#7 $$

The SS #8 Formula, named *minimum weighted bipartite matching*, considers a matching algorithm for a weighted undirected bipartite graph: given a bipartite undirected graph G = (A, B) and a weight function *w* = CS(*a*, *b*), where A and B are disjoint, and all edges link between A and B, then, a matching is a subset of edges with a minimum sum of weights and at most one edge is incident to *a* or *b* [21], i.e., a group of edges representing the most similar pairs from patient A and patient B, respectively. The Hungarian matching algorithm, also called the Kuhn-Munkres algorithm, is used to find the match [22].

### Combinations of algorithms

We use a triple such as < IC, CS, SS > to represent a combination of algorithms of three levels. To make ease of evaluations, not all the combinations were exhaustively evaluated. As the limitation of the binary code similarity has been reported in many studies, the code-level similarity #1 Formula and corresponding set-level similarity #1 to #4 Formula were not considered in this study. Combinations that have been proved to be less than optimal are excluded from this study [17, 23]. Ten representative and meaningful combinations were studied in this paper, as listed in Table 2.

**Table 2:** Selected combinations of algorithms

Triple#	IC	Code-level Similarity (CS)	Set-level Similarity (SS)
< 1,2,5>	levels(a → r)	$1-\frac{2 IC(c)}{IC(a)+ IC(b)}$	$\frac{1}{\left|A\right|+\left|B\right|}\bullet \left(\sum \limits_{a\in A}\underset{b\in B}{\min } CS\left(a,b\right)+\sum \limits_{b\in B}\underset{a\in A}{\min } CS\left(b,a\right)\right)$
< 1,2,6>			$\frac{\left({\sum}_{a\in A}\frac{1}{\left|B\right|}{\sum}_{b\in B} CS\left(a,b\right)+{\sum}_{b\in B}\frac{1}{\left|A\right|}{\sum}_{a\in A} CS\left(b,a\right)\right)}{\left|A\cup B\right|}$
< 1,2,7>			$\frac{1}{\left|A\right|\bullet \left|B\right|}\bullet \left(\sum \limits_{a\in A,b\in B} CS\left(a,b\right)\right)$
< 1,2,8>			Minimum Weighted Bipartite Matching
< 2,2,5>	$-\log \left(\frac{\frac{\left| leaves(a)\right|}{\left| subsumers(a)\right|}+1}{\left| leaves(r)\right|+1}\right)$	$1-\frac{2 IC(c)}{IC(a)+ IC(b)}$	$\frac{1}{\left|A\right|+\left|B\right|}\bullet \left(\sum \limits_{a\in A}\underset{b\in B}{\min } CS\left(a,b\right)+\sum \limits_{b\in B}\underset{a\in A}{\min } CS\left(b,a\right)\right)$
< 2,2,6>			$\frac{\left({\sum}_{a\in A}\frac{1}{\left|B\right|}{\sum}_{b\in B} CS\left(a,b\right)+{\sum}_{b\in B}\frac{1}{\left|A\right|}{\sum}_{a\in A} CS\left(b,a\right)\right)}{\left|A\cup B\right|}$
< 2,2,7>			$\frac{1}{\left|A\right|\bullet \left|B\right|}\bullet \left(\sum \limits_{a\in A,b\in B} CS\left(a,b\right)\right)$
< 2,2,8>			Minimum Weighted Bipartite Matching
< 1,3,8>	levels(a → r)	$1-{e}^{\alpha \left( IC(a)+ IC(b)-2 IC(c)\right)}\bullet \frac{e^{\beta IC(c)}-{e}^{-\beta IC(c)}}{e^{\beta IC(c)}+{e}^{-\beta IC(c)}}$	Minimum Weighted Bipartite Matching
< 1,4,8>	levels(a → r)	$\frac{IC(l)- IC(c)}{IC(l)}$	Minimum Weighted Bipartite Matching

### Prototype of subpopulations

An EMR dataset that contains age, hospital length of stay (HLOS) and diagnosis codes of 921 patients is used in this study. Four subpopulations (Table 3) are pre-defined by used of statistics or heuristic approaches. The distribution of age and HLOS of this cohort and how to select criteria are introduced in detail in the Additional file 1.

**Table 3:** Numbers of patients of four pre-defined subpopulations

Criteria	18 ≤ Age ≤ 50	Age ≥ 51
1 ≤ HLOS≤18	283	257
19 ≤ HLOS≤50	82	83

For each subpopulation, its prototype is defined as the collection of the core diagnoses of its patients. Each diagnosis has a similarity score compared with every other diagnosis. A prototype score of each diagnosis is defined as the sum of similarity scores with every other one. Core diagnoses are selected based on its prototype score which reflects the similarity of this diagnosis with all of the other diagnoses in this subpopulation.

$$ \mathrm{prototype}\ \mathrm{score}\left({d}^{\ast}\right)=\sum \limits_{d\in D} CS\left({d}^{\ast },d\right) $$

Three different code-level similarity formulas (CS #2, CS #3 and CS #4) were used to build three groups of prototypes. Then, through measuring the average of the code-level similarities between long and short HLOS subpopulations, the prototypes with more significant difference, i.e. a larger distance, at different prototype sizes are used for the further prediction task.

### HLOS prediction task

Supposing that if a targeted patient has higher similarity to one subpopulation than others, the targeted patient could be considered as one of them and share same HLOS (longer than 19 days or shorter than 18 days) with this subpopulation. Therefore, calculating the similarity between the targeted patient and the prototypes of subpopulations could work as a classifier and classify the target patient into the most similar subpopulation. Then, the average HLOS of the subpopulation is used to predict the HLOS of the target patient. In this task, different combinations of algorithms are used to measure similarity between the target patient and two subpopulations (long HLOS vs short HLOS) when using different prototype sizes. The patients with higher similarity to short HLOS subpopulations are predicted to be discharged from the hospital within 18 days. The real HLOS of the test data is used as the gold standard and to calculate the precision, recall and F-score. The F-score is the harmonic mean of precision and recall and will be used to evaluate the performance.

## Results

### Visual evaluation of IC-level algorithms

**Fig. 2:**
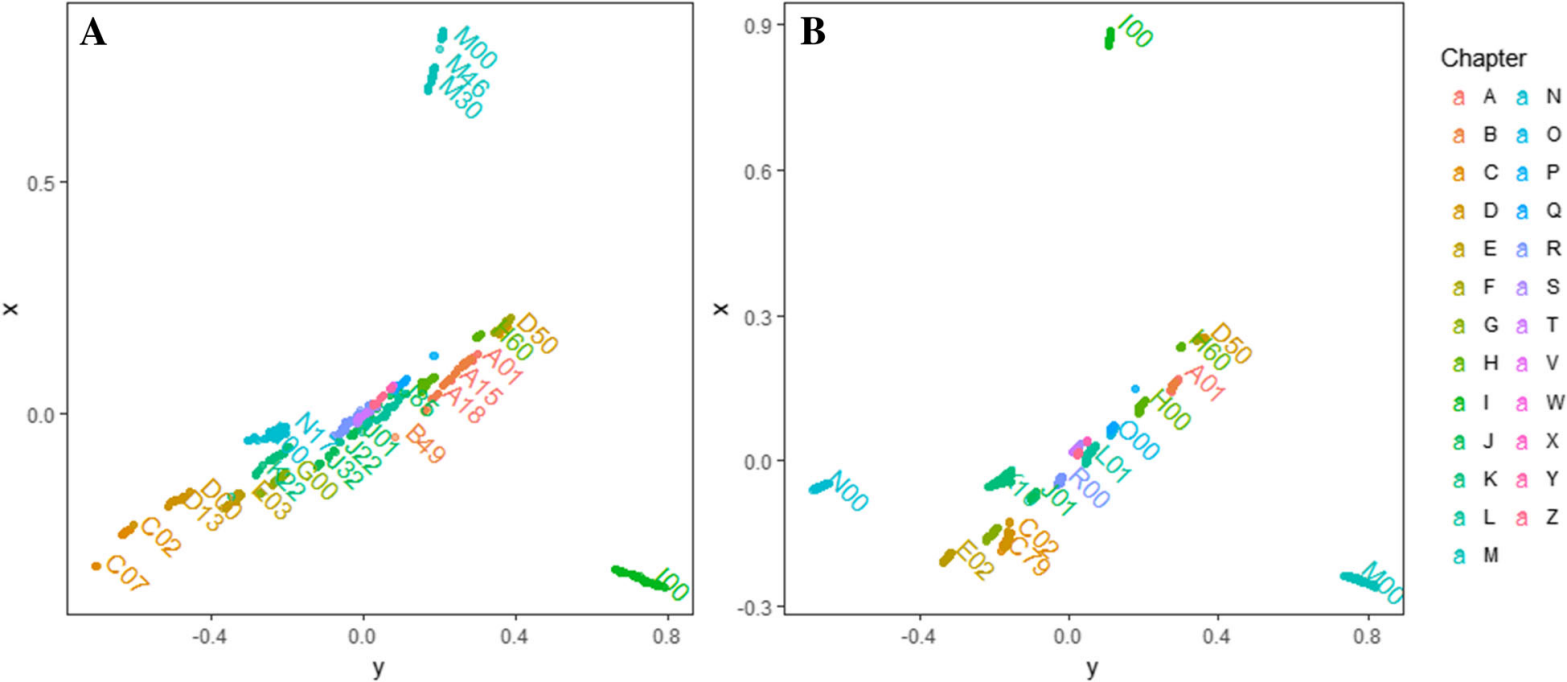
Visual comparison of two IC algorithms through the mapping distance of ICD-10 concepts. **a** IC #1 Formula was used to generate the distance matrix. **b** IC #2 Formula was used to generate the distance matrix

The path-based IC #1 Formula and the ontology-based IC #2 Formula were used to generate the distance matrix between 433 4-character ICD-10 codes that encompassed the most popular clinical diagnoses in nephrology department separately. Then, these two distance matrixes were visualized using the MDS, as shown in Fig. 2. ICD-10 codes from different subcategories were plotted with different color. It is obvious that the ontology-based IC #2 Formula was able to generate a clearer discrimination between different chapters while the path-based IC #1 Formula could separate individual codes more clearly. Therefore, the best choice depends on the concept background of the application under investigation. When the application focuses on a specific subcategory, the path-based IC #1 Formula is better and more efficient. However, the ontology-based IC #2 Formula is better suited for a more comprehensive concept background.

### Prototype of subpopulations

**Fig. 3:**
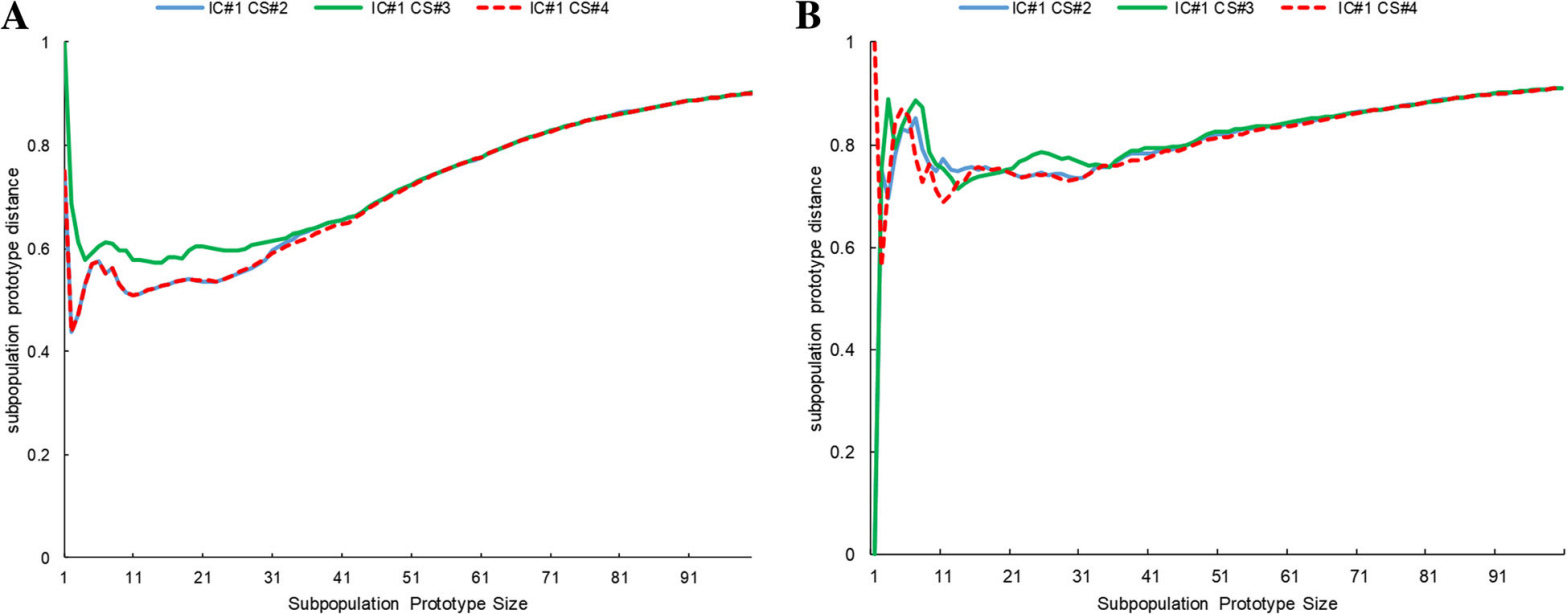
Variation of the distance between long HLOS and short HLOS subpopulation prototypes (y-axis) generated by different code-level similarity formula with respect to the size of the subpopulation prototype(x-axis). A longer distance indicates that prototypes can be separated more distinctly. **a** The comparison results of the younger population (age ≤ 50 years). **b** The comparison results of the elder population (age ≥ 50 years)

We evaluated the similarity between the long HLOS subpopulation prototype and the short HLOS subpopulation prototype with respect to the prototype size as shown in Fig. 3. The similarity between prototypes was measured by the common average of similarities of each code pairs. Basically, three groups of prototypes that were generated based on three code-level similarity formulas are comparable. When set size is larger the overlap in different prototypes tend to be larger and the difference between prototypes tend to be smaller. As the prototype score was not considered in the similarity measurement, the similarity score of two prototypes approaches to 1 when the prototype size increases. We used the area under curve and identified that the prototypes generated by CS #3 Formula (the green line in Fig. 3) achieved a better separation under different prototype sizes. This group of subpopulation prototypes was used to further evaluate all the algorithms and their combinations. The prototype score of each ICD code within each subpopulation computed by using different combinations of IC, CS can be accessed in the Additional file 2.

**Fig. 4:**
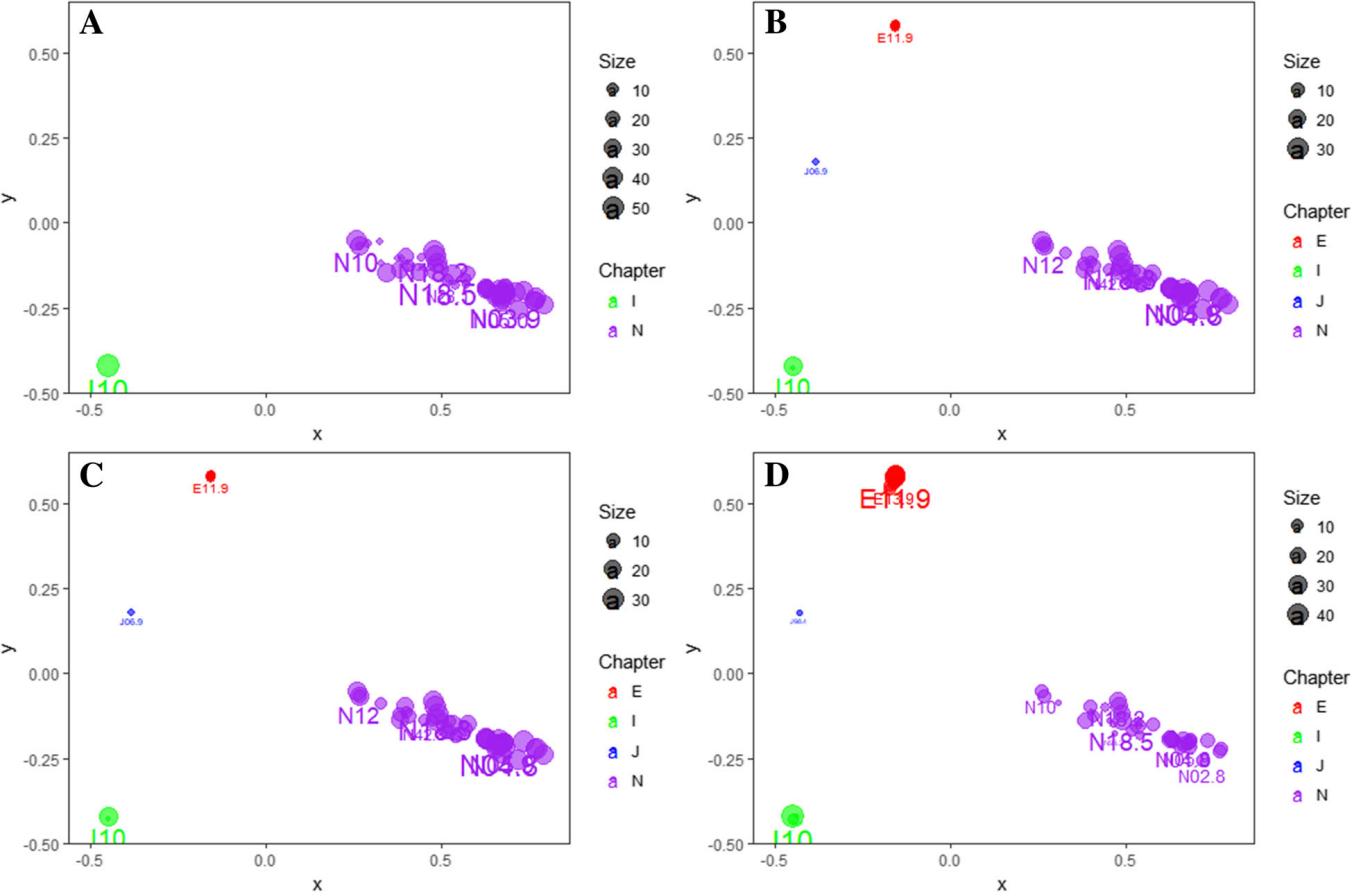
The scatter plots of diagnoses prototypes of four subpopulations used for evaluation. The size of the circle is a relative scaled prototype score of the diagnosis code in the subpopulation. The color of the circle depends on the first letter of the ICD-10 code. **a** A subpopulation younger than 50 years old with HLOS shorter than 18 days. **b** A subpopulation younger than 50 years old with HLOS longer than 19 days. **c** A subpopulation older than 50 years old with HLOS shorter than 18 days. **d** A subpopulation older than 50 years old with HLOS longer than 19 days

For a closer inspection of the prototypes of four subpopulations, the top 50 diagnoses concepts from each subpopulation prototype were plotted using their distance and scaled prototype score in the subpopulations (as shown in Fig. 4). As expected, each subpopulation prototype was dominated by the diseases of kidney and urinary systems (the purple circles in Fig. 4). A difference was observed in the chronic conditions such as diabetes and hypertension (the pink and green circles in Fig. 4). Basically, these chronic complications had higher weights in the elder and long HLOS subpopulations. Hypertension was very common in all of the subpopulations with kidney disease, as it is one of the leading causes of kidney failure. However, diabetes was seemingly associated with the long HLOS in both elder and young subpopulations. There were also slight differences among the groups of kidney diseases. The Additional file 2 details the prototype score of each ICD code within each subpopulation; a full list of 95 unique 4-character ICD codes, the adjacency matrix and coordinates of each code is provided.

### Performance of algorithms

To measure how well various combinations performed at predicting whether a patient will be discharged from the hospital within 18 days, we designed a classifier that a target patient would be classified into a subpopulation if he had a higher similarity score with the prototype of this subpopulation than another. The similarity scores of each patient and each prototype are listed in the Additional file 3. The performance of algorithm combinations is evaluated by assessing the ability of the classifier to determine whether test patients had higher similarity to the prototype of short-term cohort.

**Fig. 5:**
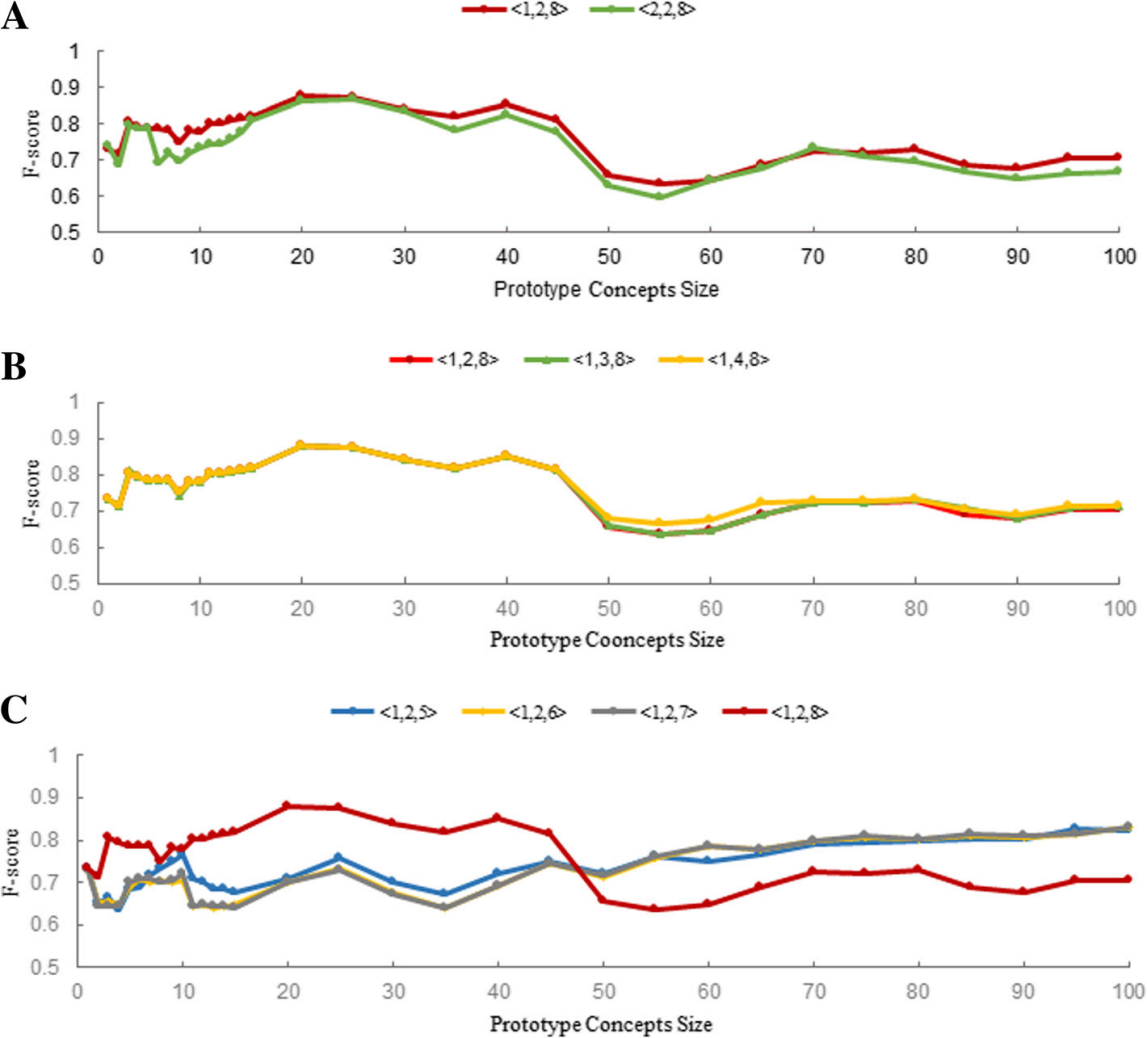
Variation of the algorithm performance (y-axis) with respect to the size of the retrieved concepts list forming the prototype (x-axis). **a** The IC-level comparisons. **b** The code-level comparisons. **c** The set-level comparisons

The effect of a single variable on the prediction performance by controlling other effect factors constant is analyzed respectively, e.g., CS algorithm and SS algorithm are constant when IC algorithms are compared. For each combination, we took the similarity scores obtained by all patients in the test set (i.e. 216 patients) and measured the precision, recall and F-score. In Fig. 5, only F-score is shown (for details please refer to Additional file 4). The results regarding precision and recall can be found in the Additional file 5. The details of the similarity score between each case and each prototype with various set sizes computed by using different combinations of IC, CS and SS can be found in the Additional file 3.

#### IC-level evaluation

As mentioned above, IC performance visualization analysis, the ontology-based IC #2 Formula had better discriminative power at the chapter-level compared to the path-based approach. However, the IC #1 Formula was able to separate different concepts under the same subcategory more distinctly. In the prediction task, most of the concepts were from the same subcategory, as shown in Fig. 4. Therefore, the result shown in Fig. 5a confirmed that the performance of the IC #1 Formula was superior to that of the IC #2 Formula in a narrow concept background.

#### Code-level evaluation

As shown in Fig. 5b, three code-level algorithms were compared in this task. The code-level algorithm #3 Formula, which is more computation-intensive, does not provide an obvious promotion in performance. However, it provides two parameters, α and β, which will be helpful when the taxonomy is more complicated, making it possible to control and adjust the performance. We did not optimize these two parameters in this study. The other two code-level similarity algorithms achieved a similar performance. CS #4 Formula, as a simplified CS #2 Formula, was sometimes able to achieve appreciably better results.

#### Set-level evaluation

As shown in Fig. 5c, the performance of different set-level similarity algorithms was not consistent with each other. SS #8 algorithm was the most vulnerable one. It performs better when the prototype concepts size is relatively smaller than 45. However, when the prototype concept size increases, the prototype of different subpopulations will have approximately the same components as all these subpopulations that belongs to the same clinical department. Therefore, minimum weighted bipartite matching lost its discrimination power for classification. It does not consistently outperform the other three set-level algorithms. The others are relatively stable and improved gradually as the prototype concept size increased. The same results were confirmed when using the IC #2 as the controlled IC algorithm. More details are accessible in the Additional file 5.

## Discussion

### Balance between efficiency and effectiveness

The overall performance of an algorithm is measured in terms of efficiency and effectiveness. Regarding the information content methods, the IC #2 Formula costs more in data preprocessing time than the IC #1 Formula since the former one needs to count the leaves of the child tree of every node in the ICD tree and we calculated the IC value of every code in advance to accelerate computation in this test. With the preprocessing completed, the time cost of real-time operation of the two IC methods makes very little difference. Regarding the code-level similarity methods, the CS #3 Formula uses five exponents, and the efficiency is somewhat poor. However, in the set-level similarity methods, the time complexity of the minimum weighted bipartite matching (the SS #8 Formula) method is O(*n*^3^), while for the other three it is O(*n*^2^), where *n* is the count of concepts, which is a significant difference, especially when the set size is large. Efficiency and effectiveness are central terms in assessing algorithm performance, yet the challenge for patient similarity is to balance efficiency with effectiveness in practice.

### Concept set sizes and scenarios

The concept set size not only influences the computational load but also impacts the algorithm performance appreciably in some scenarios. The major performance difference among these algorithms combinations are from the set-level similarity computation. It is due considering the exponential pairs when the size increases. The average of the similarity scores will be diluted if most of them are dissimilar. The imbalance of the two compared concept set size could also bring similar problems. In this study, the prototype size changed from 1 to 100. The performance of different combinations was not stable when the prototype size was below 20. However, most average approach methods will become stable in the later stage.

**Fig. 6:**
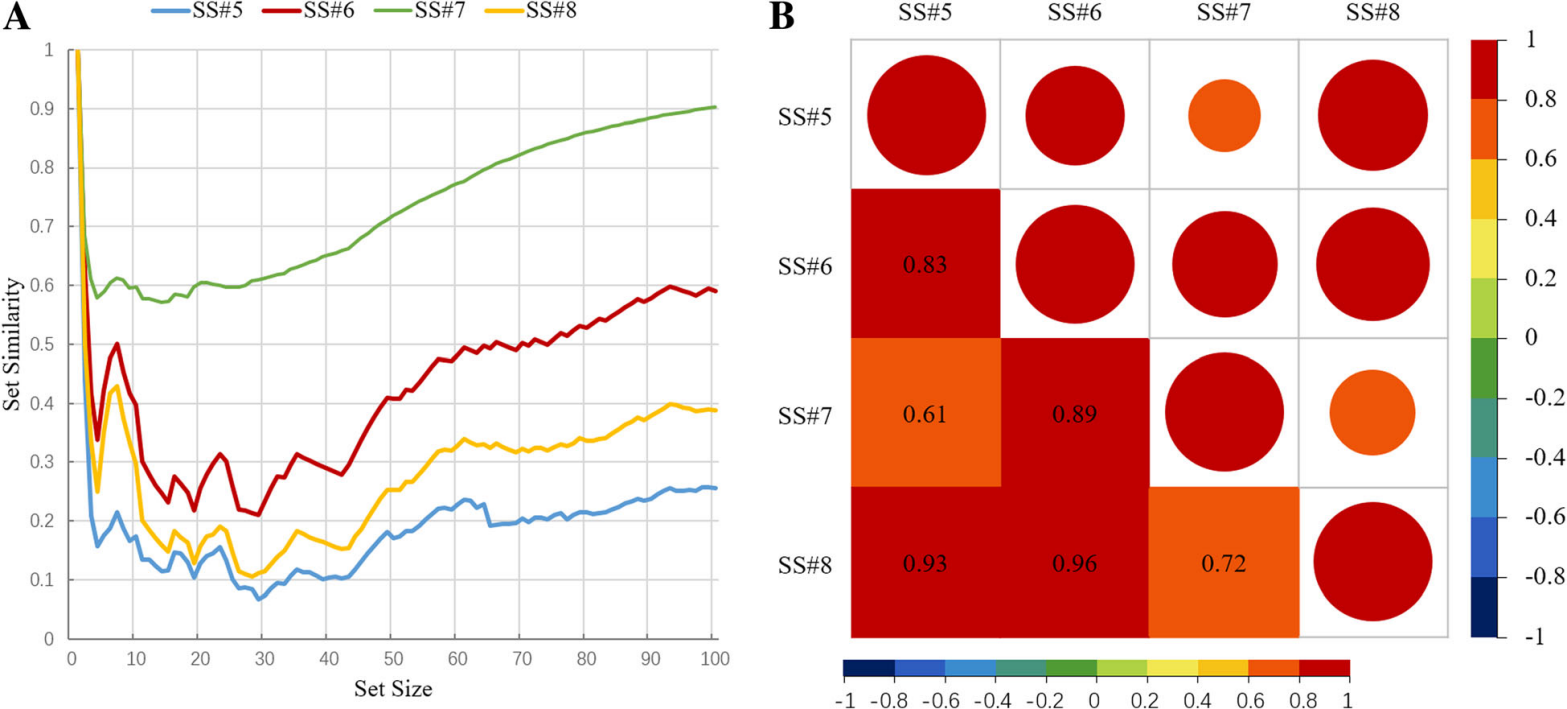
Correlation between set-level methods. **a** Using four set-level similarity algorithms to measure the distance of two prototypes with different prototype set sizes. **b** The correlation between each two SS methods

The concept set background should be considered when choosing the algorithm as well. The two IC-level algorithms have different applicable scenarios. The set-level similarity algorithms also have different responses to the diversity of the set contents. Figure 6a shows the different set-level algorithms’ responses to the change of prototype size. In Fig. 6a, the similarity score between the prototype (age 18~50, HLOS 1~17) and the prototype (age 18~50, HLOS 18~50) used in the evaluation was computed by using different set-level algorithms at different set sizes. A smaller set-level score means higher similarity. Although the responses are highly correlated, the response ranges are obviously different. Figure 6b shows the exact pair-wise correlation value of the four SS algorithms. A larger circle and a redder color represent higher correlation.

### The minimum weighted bipartite matching approach

The minimum weighted bipartite matching method finds the best matches in an undirected bipartite graph. This method has not been well recognized to measure the similarity of concept sets in published studies. However, it shows better performance for similarities at the set level in the evaluation. It was also confirmed when using different IC levels or code-level algorithms. Minimum weighted bipartite matching tries to find the most similar diseases and ignores the redundant ones, whereas the average approaches (SS#6 and #7 Formulas) dilute the similarity and magnify the dissimilarity, which makes the similarity score of sets approach to 1 when the set size increases.

In Fig. 6, SS #5 Formula, which uses the average of the most similar pairs from each set, showed similar features to preserve the set-level similarity with the minimum weighted bipartite matching. However, when the sizes of two sets are unbalanced, where the patient set size is approximately 4 and the prototype size ranges from 1 to 100, the most similar pairs from the larger set can dilute the whole similarity. Therefore, in our evaluation, < 1,2,5 > only shows a slightly better performance compared to < 1,2,6 > and < 1,2,7 > before the turning point which is same as the turning points of < 1,2,8 > when two subpopulations are almost identical.

In a summary, the minimum weighted bipartite matching method is better suited to measuring set similarity especially when the sizes of two sets are large or unbalanced. But it is not an efficient choice when the set size is small and balanced.

### Taxonomic concept-based patient similarity

A taxonomy-based similarity measurement is suitable for other situations in addition to the diagnoses-related similarity. It could be used in the field of other standard clinical terms, e.g., MeSH [24], ICD-10-CM [25], and ATC etc. These standard clinical terms are hierarchical and taxonomic and are arranged in a tree structure. These codes support both the path-based IC method and the ontology-based IC method. The algorithms to compare a pair of codes and two sets of codes are similar to the algorithms for diagnoses introduced in this paper. For instance, Çelebi [26] defined a drug therapeutic similarity as the average of the Jaccard similarity coefficient of ATC codes of all levels. Patients with a superficial injury (ICD-10 T14.0), open wound (ICD-10 T14.1) of different body regions, e.g., scalp (MeSH A01.456.810), ear (MeSH A01.456.313), and neck (MeSH A01.598), can be compared by using the taxonomic codes.

## Conclusions

Patient similarity is a big data tool to improve the precision of predicting future health states of patients, which has important clinical significance and paves the way to personalized medicine. The accumulating raw data in the health care domain can be used to assess patient similarity by leveraging the taxonomic standard codes. In this paper, we considered diagnoses with ICD-10 as an example and compared related various methods for IC of taxonomic concepts, code-level similarity and set-level similarity with new proposed algorithms. Each algorithm at different levels was evaluated through data visualization and a prediction task. Our research suggests that 1) A better IC calculation algorithm depends on the concept background. The efficient path-based IC is also effective when the application focuses on a narrow subdomain. However, it is not as good as ontology-based IC to separate subcategories in a more comprehensive scenario. 2) All of the IC-based code-level similarity algorithms are comparable to each other. 3) The minimum weighted bipartite matching algorithm performs better to measure the set-level similarity at different set sizes and for unbalanced set sizes.

## Additional files

Additional file 1: Data Interpretation and Subpopulation Criteria. Details of the process of collecting and filtering data and of the criteria of dividing subpopulation. (DOCX 35 kb)

Additional file 2: Subpopulation prototype. The prototype score of each ICD code within each subpopulation, a full list of 95 unique 4-character ICD codes, the adjacency matrix and coordinates of each code. (XLSX 75 kb)

Additional file 3: Similarity Score of Each Patient and Prototypes. Details of similarity score between each patient and each prototype with various set size computed by using different combinations of IC, CS and SS. (XLSX 5114 kb)

Additional file 4: Prediction results. The raw data of prediction results. (XLSX 44 kb)

Additional file 5: Supplemental results not included in the main text. (DOCX 154 kb)

## Abbreviations

CS: Code-level similarity
EMR: Electronic Medical Records
HLOS: Hospital Length of Stay
IC: Information Content
LCA: Least common ancestor
SS: Set-level Similarity
