# Supplementary material for: Using the distance between sets of hierarchical taxonomic clinical concepts to measure patient similarity
Source: BMC Med Inform Decis Mak. 2019 Apr 25;19:91. doi: 10.1186/s12911-019-0807-y (PMC6485152; doi:10.1186/s12911-019-0807-y)
Supplement: Supplementary file 1 — Data Interpretation and Subpopulation Criteria. Details of the process of collecting and filtering data and of the criteria of dividing subpopulation. (DOCX 35 kb) [file 12911_2019_807_MOESM1_ESM.docx]

**Data Interpretation and Subpopulation Criteria**

**Data interpretation**

We obtained the EHR dataset from a third-level grade-A hospital, Shanxi Dayi Hospital. The data set contains 1666 inpatients from the nephrology department between April 2014 and May 2015. We built a case base including demographics, HLOS, and discharge diagnoses (with 4-character ICD-10 subcategories). The dataset covers the entire adult age spectrum ranging between 7 and 90 years. To assess the pros and cons of each method and enhance credibility, we have developed some criteria for screening patients.

1. Patients represented in the dataset paid at least ¥200/day for treatment and hospitalization. The requirement was set to ensure that each case received the effective and appropriate treatment that they needed and deserved.
2. Patients with more than 2 missing ICD codes of diagnoses were removed for the reason that hospitals in China usually suffer from the contradiction between a shortage of skilled coders and an excess of hospital patients, and coders may neglect or skip some discharge diagnoses to save time and fulfill their duties.
3. In China the age of majority is 18. Infants and juveniles (<18 years), and very-long-term (>50 days) inpatients were removed from the datasets to avoid biases.

Finally, there were 921 remaining inpatients and 499 distinct clinical diagnoses, within which 705 randomly selected distinct inpatients and 433 corresponding distinct diagnoses are used to evaluate algorithms for IC of taxonomical concepts and build prototypes and others are used as test set to predict HLOS. The distribution figures of age and HLOS of 921 inpatients are shown below.


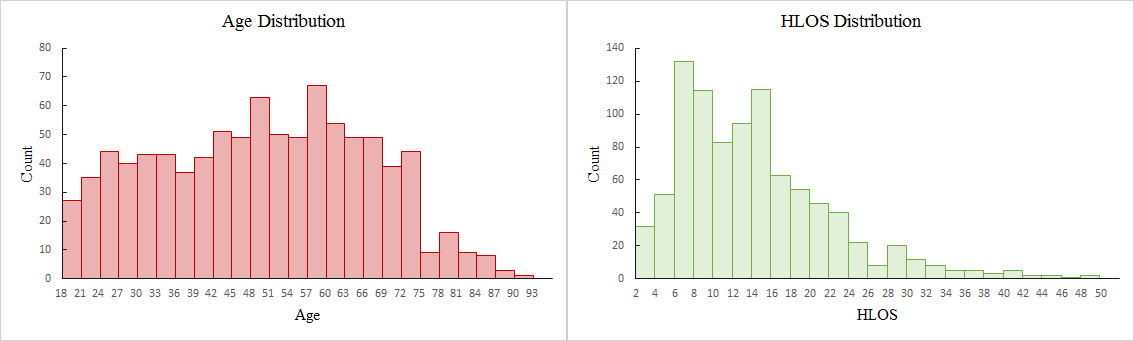


Supplemental Figure 1 Distribution of age and HLOS of study cohort

**The subpopulation criteria**

Patients were divided into four cohorts stratified by their age (18-50 years and 51-100 years) and HLOS (1-18 days and 19-50 days). The reason for the stratification of age is that the diseases of infants, juveniles, middle-aged adults, and older patients differ. 50 years old was chosen as a watershed since that worldwide most women enter menopause between 49 and 52 years of age [1]. Regarding HLOS, the K-means algorithm was used to identify relatively homogeneous groups of cases based on the characteristic, HLOS. The number of clusters was 2. The maximum number of iterations was 10. IBM SPSS Statistics K-means Clustering tool was used to process the data. The numbers of patients of four pre-defined subpopulations are presented in the following table.

Supplemental Table 1: numbers of patients of four pre-defined subpopulations

| Criteria | 18≤Age≤50 | Age≥51 |
| --- | --- | --- |
| 1≤HLOS≤18 | 283 | 257 |
| 19≤HLOS≤50 | 82 | 83 |

**Reference**

[1] Takahashi TA, Johnson KM. Menopause. Med Clin 2015;99:521–34. doi:10.1016/j.mcna.2015.01.006.
