# Supplementary material for: Using the distance between sets of hierarchical taxonomic clinical concepts to measure patient similarity
Source: BMC Med Inform Decis Mak. 2019 Apr 25;19:91. doi: 10.1186/s12911-019-0807-y (PMC6485152; doi:10.1186/s12911-019-0807-y)
Supplement: Supplementary file 5 — Supplemental results not included in the main text. (DOCX 154 kb) [file 12911_2019_807_MOESM5_ESM.docx]

**Precision and Recall of Classification**

***Precision***

The performance of matching patients with different prototypes was used to assess the IC methods, CS methods and SS methods. The precision is shown below.


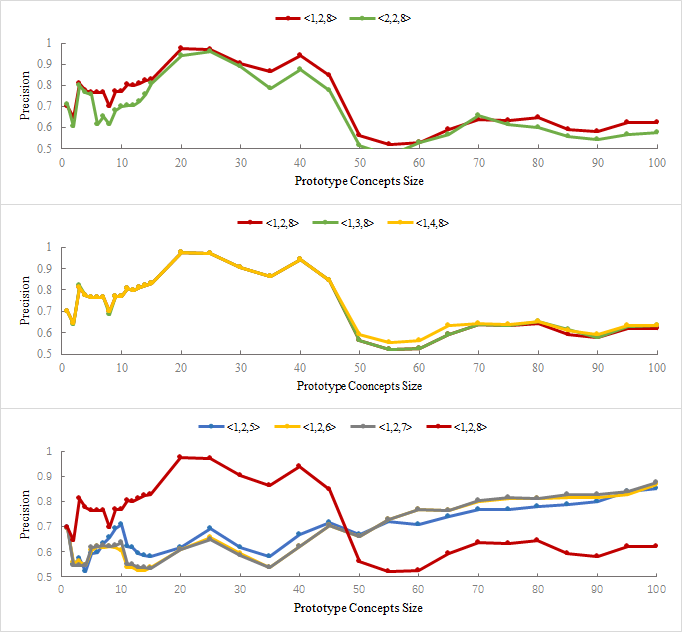


Supplemental Figure 2 The precision of the performance of classification.

***Recall***

The performance of matching patients with different prototypes was used to assess the IC methods, CS methods and SS methods. The recall is shown below.


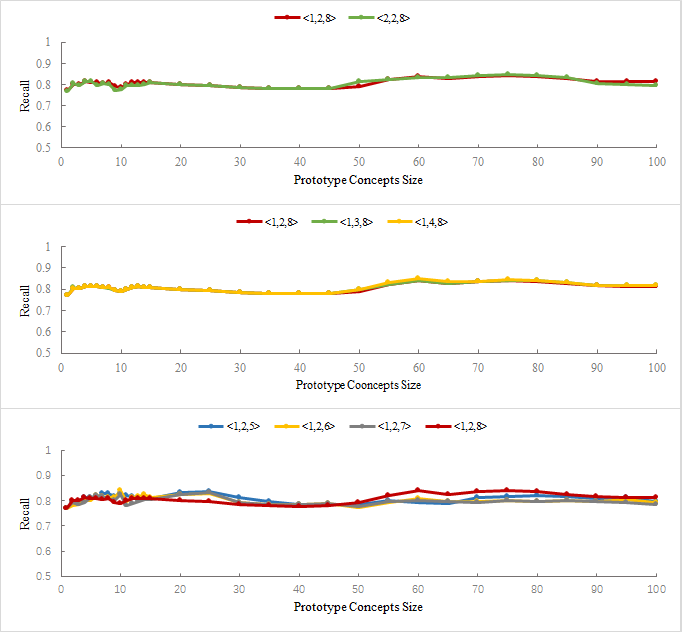


Supplemental Figure 3 The recall of the performance of classification.

**Performance of Combinations with Taxonomy-based IC**

When IC is computed based on taxonomy (IC#2 Formula), the performance of matching patients with different prototypes and predicting HLOS length is as shown below. Thess results also confirmed the result shown in the main text.


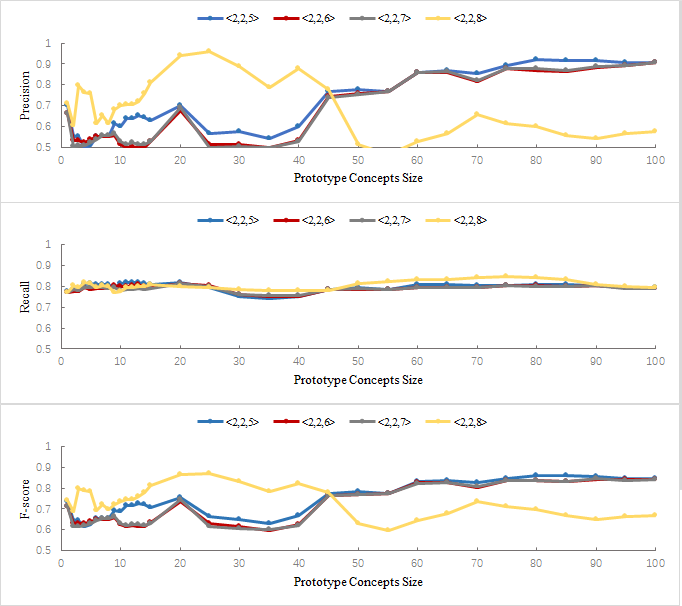


Supplemental Figure 4 Performance of Combinations using Taxonomy-based IC
